# Method Validation for Simultaneous Quantification of Olmesartan and Hydrochlorothiazide in Human Plasma Using LC-MS/MS and Its Application Through Bioequivalence Study in Healthy Volunteers

**DOI:** 10.3389/fphar.2019.00810

**Published:** 2019-07-23

**Authors:** Arvind Kumar, Surya Prakash Dwivedi, Tulika Prasad

**Affiliations:** ^1^School of Biotechnology, IFTM University, Moradabad, India; ^2^Advanced Instrumentation Research & Facility (AIRF) and Special Centre for Nano Sciences (SCNS), Jawaharlal Nehru University, New Delhi, India

**Keywords:** hypertension, olmesartan, hydrochlorothiazide, LC-MS/MS, pharmacokinetics, bioequivalence

## Abstract

A new, simple, sensitive, selective, rapid, and high-throughput liquid chromatography-tandem mass spectrometry (LC-MS/MS) method has been developed and validated for simultaneous quantification of Olmesartan and hydrochlorothiazide in human plasma. Simple liquid–liquid extraction procedure was applied for plasma sample pretreatment using a mixture of diethyl ether and dichloromethane, as an extraction solution. Analytes were separated on UNISOL C18 150*4.6 mm, 5 µm column using methanol, and 2 mM ammonium acetate pH 5.5 (80:20, v/v) as a mobile phase and detected by electrospray ionization in the multiple reaction monitoring (MRM) mode. The mass transition ion pairs were followed in negative ion mode as m/z 445.20 → 148.90 for Olmesartan; m/z 451.40 → 154.30 for Olmesartan D_6_ and m/z 295.80 → 205.10 for hydrochlorothiazide; m/z 298.90 → 206.30 for hydrochlorothiazide ^13^C D_2_. The method showed excellent linearity (*r* ^2^ > 0.99) over the concentration range of 5.002–2,599.934 ng/ml for Olmesartan and from 3.005 to 499.994 ng/ml for hydrochlorothiazide. Precision (% CV) and accuracy (% bias) for Olmesartan were found in the range of 3.07–9.02% and −5.00–0.00%, respectively. Precision (% CV) and accuracy (% bias) for hydrochlorothiazide were found in the range of 3.32–8.21% and 1.99–3.80%, respectively. This as developed novel and high-throughput liquid–liquid extraction bioanalytical method has substantial innovative value with the benefits of cost effectiveness, good extraction efficiency, shorter analysis run time, low organic solvent consumption, and simpler procedure over the previously reported solid-phase extraction method. The application of this method in pharmacokinetic studies was further demonstrated successfully through a bioequivalence study conducted on healthy human subjects, following oral administration of combined formulation of Olmesartan medoxomil and hydrochlorothiazide in fixed-dose tablet.

## Introduction

Hypertension or high blood pressure, sometimes called arterial hypertension, is a chronic medical condition in which the blood pressure in the arteries is elevated due to the higher force exerted by blood against the wall of the blood vessels (Mancia et al., 2013). According to the report by the Centers for Disease Control and Prevention (CDC), hypertension represents one of the most prevalent pathology worldwide. Nearly 1.13 billion people or ∼26% of the adult population of the world were reported with hypertension as per the report of World Health Organization (WHO). It is common in both developed (333 million) and undeveloped (639 million) countries as reported by Kearney et al. (2005).

Olmesartan medoxomil and hydrochlorothiazide are reported as the two most preferred drugs of choice for combination therapy of hypertension (Chrysant et al., 2004; Greathouse, 2006; Bramlage et al., 2013). Olmesartan medoxomil has the better antihypertensive effect when treatment is combined with diuretics (Zhang et al., 2017) and is described chemically as the (5‐methyl‐2‐oxo‐1,3‐dioxol‐4‐yl) methyl ester of 4‐(1‐hydroxy‐1‐methylethyl)‐2‐propyl‐1‐{[20‐(1*H*‐tetrazol‐5‐yl) [1,10‐biphenyl]‐4‐yl]methyl}‐1*H*‐imidazole‐5‐carboxylic acid. After oral administration of the prodrug, Olmesartan medoxomil ester moiety, there occurs a rapid cleavage of the ester moiety *via* endogenous esterase to result in the release of the active metabolite i.e., Olmesartan (OLM). OLM is known to be a selective angiotensin AT1 receptor blocker (Sada and Mizuno, 2004). Hydrochlorothiazide (HCTZ) is a thiazide diuretic of benzothiadiazine, chemically described as 6-chloro-1,1-dioxo-3,4-dihydro-2H-1λ^6^,2,4-benzothiadiazine-7-sulfonamide. HCTZ acts by inhibiting sodium re-absorption in the renal tubule and increasing the rate of urinary excretion of sodium and water, which then leads to reduction in cardiac output and blood volume. Low plasma rennin activity is often associated with hypertension, and HCTZ is reported to be effective for treatment of individuals with low rennin hypertension and may require a longer treatment regime (Goswami et al., 2008). Therefore, for proper management of hypertension, it is recommended to provide a combination therapy of HCTZ along with an anti-hypertensive drug (Zanchetti, 2003).

A number of methods have been reported for analysis of OLM and HCTZ simultaneously, separately or in combination with other drugs in pharmaceutical dosage forms which involves various techniques—namely, spectrophotometer (Wankhede et al., 2009; Rote and Bari, 2010), HPLC (Wankhede et al., 2009; Kamble et al., 2010; Rao et al., 2011; and Doshi et al., 2012), and HPTLC (Shah et al., 2007; Kamble et al., 2010), but these methods cannot be applied to the clinical pharmacokinetic studies. A few LC-MS/MS methods are reported for quantitation of OLM and HCTZ, separately or in combination with other drugs (Yu et al., 2006; Goswami et al., 2008; Vaidya et al., 2008; Rajasekhar et al., 2009; Shah et al., 2009; Tutunji et al., 2009; Gao et al., 2010; Sengupta et al., 2010; Tutunji et al., 2010; Bharathi et al., 2012; Gadepalli et al., 2014), but these are not suitable for the simultaneous quantitation of OLM and HCTZ. However, few methods are also reported for the simultaneous estimation of OLM and HCTZ in human plasma by LC-MS/MS (Liu et al., 2010; Kumar et al., 2012; Kumar et al., 2014; Sable et al., 2016), but all of them have applied the solid-phase extraction (SPE) method for drug extraction from plasma. The SPE method is quite tedious and costly due to the inevitable use of SPE cartridges. This results in a relatively long extraction time and increases the burden on the laboratory budget. Therefore, this method has been rendered tedious and time-consuming especially for those clinical studies with a considerable sample size and could not turn into the method of choice for pharmacokinetic analysis in clinical studies. The method reported by Liu et al. (2010) was validated for linearity range between 1 and 1,000 ng/ml for OLM in human plasma, which is not sufficient for the pharmacokinetic evaluation of 40 mg OLM dose with anticipated *C*_max_ of approximately 1,350 ng/ml. The method by Sable et al. (2016) was not sensitive enough to evaluate the pharmacokinetics of OLM and HCTZ in human plasma, i.e., lower limit of quantification quality (LLOQ) was 32.32 ng/ml for OLM and 5.12 ng/ml for HCTZ. This method was yielding lower recovery. The report by Kumar et al. (2014) displayed good sensitivity and dynamic linearity range but suffered from the longer analysis run time (5 min) and used tedious and expensive sample pre-treatment method i.e., solid-phase extraction. Solvent consumption was also higher in the sample pre-treatment, which is likely to increase the organic load in the environment. Sample pre-treatment is the major part in the analysis of drugs from biological samples and more than 50% of cost, labor participation, and errors are associated with the sample pre-treatment. Therefore, it is always advisable to make the sample pre-treatment process as simple, robust, and cost-effective as possible, without compromising the selectivity, sensitivity, precision, and accuracy. In the light of this background, the development of a simple, cost effective, and rapid bioanalytical method for simultaneous quantification of OLM and HCTZ certainly merits attention for high-throughput and faster evaluation of pharmacokinetics of fixed dose combined formulation, suitable for even a large sample size.

There are reports (Gao et al., 2010; Sengupta et al., 2010; Bharathi et al., 2012) which give insight that these drugs can be extracted from plasma by liquid–liquid extraction (LLE) which is simple, robust, and cost-effective. The aim of this study was to develop and validate a simple, sensitive, selective, rapid, and high-throughput LC-MS/MS assay employing LLE for sample preparation for the simultaneous determination of OLM and HCTZ in human plasma, in accordance to USFDA guidelines (U.S. Food and Drug Administration, 2001). To the best of our knowledge, this is the first simultaneous extraction of OLM and HCTZ from human plasma samples by applying the LLE method instead of solid-phase extraction. Furthermore, the applicability of this method in pharmacokinetic studies was demonstrated by conducting a bioequivalence study on healthy human subjects.

## Material and Methods

### Chemicals

Working standards of Olmesartan acid (OLM) and OLM D_6_ (OLM D_6_) were procured from VIVAN Life Sciences (Thane-Mumbai, India) and Simson Pharma (Dahisar-Mumbai, India), respectively. Working standard of HCTZ was also procured from VIVAN Life Sciences and HCTZ ^13^C D_2_ (HCTZ ^13^C D_2_) was purchased from Splendid Lab (Pune, India). OLM D_6_ and HCTZ ^13^C D_2_ were used as internal standards (IS) for OLM and HCTZ, respectively. Methanol was purchased from Merck. Drug-free human plasma with ethylene diamine tetra acetic acid (K_2_EDTA) was purchased from Laxmi Sai Clinical Labs (Hyderabad, India). Formic acid (SQ grade) and ammonium acetate (Excela R grade) were purchased from Qualigens Fine Chemicals (Mumbai, India). Diethyl ether and dichloromethane were obtained from S.D. Fine Chemicals Ltd. (Mumbai, India). Milli-Q water with resistivity of 8.2 milliohm at 25°C and total organic carbon (TOC) ≤500 ppb was used from the in-house Milli-Q water purifying system (Millipore, SAS, Molsheim, France).

### Instrumentation

Liquid chromatography mass spectrometer (LC-MS/MS) analysis was performed in multiple reactions monitoring (MRM) mode using Mass Spectrometer (API 4000 from Applied Biosystems MDS SCIEX, Toronto, Canada) interfaced with high-performance liquid chromatography system (Prominence 20 AD from Shimadzu Corporation, Kyoto, Japan). Turbo electrospray ionization (ESI) source was used as the interface in negative ionization mode. The chromatographic data were acquired and processed using computer-based Analyst Software version 1.4.2 of Applied Biosystems and Watson LIMS (Laboratory Information Management System) version 7.4 SP3.

### Preparation of Stock Solutions, Calibration Curve (CC) Standards, and Quality Control (QC) Samples

The primary stock solutions of OLM (5 mg/ml), HCTZ (1 mg/ml), OLM D_6_ (1 mg/10 ml), and HCTZ ^13^C D_2_ (1 mg/10 ml) were separately prepared in methanol. Further stock dilutions for both analytes i.e., OLM and HCTZ were prepared for CC and QC from primary stock solutions by appropriate dilutions with methanol and Milli-Q water in the ratio 50:50 (v/v).

The above prepared CC dilutions were spiked in interference free K_2_EDTA plasma to yield a set of eight non-zero CC standards each for OLM and HCTZ, respectively. The eight CC standards for OLM included concentrations of 5.002 ng/ml, 10.004 ng/ml, 299.512 ng/ml, 599.025 ng/ml, 1,198.050 ng/ml, 1,633.358 ng/ml, 2,129.568 ng/ml, and 2,599.934 ng/ml. The respective concentrations of the eight CC standards for HCTZ were 3.005 ng/ml, 6.010 ng/ml, 62.599 ng/ml, 125.199 ng/ml, 1,198.050 ng/ml, 1,633.358 ng/ml, 2,129.568 ng/ml, and 2,599.934 ng/ml.

Respective samples for quality control were prepared by spiking QC dilutions in interference free K_2_EDTA plasma to yield final concentrations of lower limit of quantification quality control (LLOQ-QC), lower quality control (LQC), middle quality control (MQC), and higher quality control (HQC) for OLM and HCTZ. The concentrations used for OLM were 5.006 ng/ml (LLOQ QC), 13.906 ng/ml (LQC), 1,198.829 ng/ml (MQC), and 2,131.831 ng/ml (HQC).

For HCTZ, the concentrations of the respective QCs were 3.008 ng/ml (LLOQ QC), 8.076 ng/ml (LQC), 252.367 ng/ml (MQC), and 419.392 ng/ml (HQC). Internal standard dilution mixture was prepared in methanol: Milli-Q water (50:50 v/v) containing OLM D_6_-2,000.00 ng/ml and HCTZ ^13^C D_2_-2,800.00 ng/ml.

The CC standards and QC samples mentioned above were stored in deep freezer at ultra-low temperature of −65°C ± 10°C until further analysis. The primary stock solution and dilutions were stored at 2–8°C.

### Chromatography Conditions

The solvents used for mobile phase consisted of methanol and buffer solution A (2 mM ammonium acetate pH 5.5 adjusted with acetic acid) in the ratio of 80:20 (v/v). The mobile phase, degassed in an ultrasonicator and filtered through 0.2-µm filter, was used at a flow rate of 0.8 ml/min. The processed samples were subsequently loaded in the auto-sampler set at the temperature of 5°C. A sample volume of 15 µL was injected onto the column for analysis. The column used was UNISOL^®^ C18 150*4.6 mm, 5 µm was obtained from Agela Technologies, and the column oven temperature was maintained at 35 ± 2°C.

### Sample Preparation and Extraction Method

A novel and high-throughput liquid–liquid extraction (LLE) method was developed for sample preparation. Out of the different extraction solvents (namely, *n*-hexane, diethyl ether, methyl *tert*-butyl ether (MTBE), dichloromethane, and ethyl acetate and their mixtures in varying composition) tried, the mixture consisting of diethyl ether and dichloromethane in the ratio 70:30 (v/v) was found to be the most suitable for the extraction of OLM and HCTZ and their respective IS, wherein no matrix interference was observed.

Sample processing was carried out under sodium vapor light. The set of CC standards and appropriate QC samples were taken out from the deep freezer and thawed at room temperature. Then, 300 μl of plasma samples were added into RIA vials. 50 μl of internal standard dilution mixtures (OLM D_6_-2,000.00 ng/ml and HCTZ ^13^C D_2_-2,800.00 ng/ml) were added into these RIA vials (except blank sample), and the vials were uniformly vortexed. Further, 100 μl of buffer solution B, formic acid:Milli-Q water (2:98, v/v), was added to all samples and vortexed for approximately 1 min.

The analytes and their respective IS were extracted from plasma by using an extraction solution which is the mixture of diethyl ether and dichloromethane (70:30, v/v). The samples were vortexed for 10 min after addition of 2.5 ml of the extraction solution. This was followed by the centrifugation of the samples at 4°C for 5 min at 4,500 rpm. The samples were then flash frozen for 2 min approximately, and the supernatant collected were evaporated to dryness at 50°C in nitrogen evaporator (at constant pressure). Reconstitution of residue was carried out with 600 μl of mobile phase, which was then transferred into HPLC vials for further analysis on LC-MS/MS.

### Method Validation

The validation of the method was carried out for sensitivity, selectivity, matrix effect, linearity, CC standards and QC samples, precision, and accuracy batches. The results obtained for diverse range of stabilities (i.e., stock solution and stock dilution stabilities at room temperature and refrigerator temperature, freeze-thaw stability, autosampler stability, long-term stability at −65°C ± 10°C, re-injection reproducibility, reagent stability, dry extract stability, wet extract stability, bench top stability, extended bench top stability, blood stability, lipemic and hemolyzed plasma stability), recovery, dilution integrity, robustness, ruggedness, ion suppression through infusion, extended batch verification, and effect of potentially interfering drugs (PID) were found to fulfill the pre-set acceptable criteria of the USFDA guidelines (U.S. Food and Drug Administration, 2001).

### Selectivity and Isotopic Interference

Four lots of lipemic, four lots of hemolyzed, and eight lots of normal plasma which contained K_2_EDTA (potassium salt of ethylene Diamine tetra acetic acid) as an anticoagulant were used for assessing the selectivity of the method. The plasma lots were evaluated to ensure no significant interference at respective retention time (RT) of analyte and IS. The interference was evaluated in each blank matrix by comparing with the response of respective LLOQ samples (blank matrix spiked with LLOQ dilution). Isotopic interference was also assessed to ensure no significant interference at the RT of analyte in blank containing IS. Sensitivity of the method was determined by evaluating signal to noise ratio in LLOQ sample in order to ensure more than five times response in LLOQ as compared to the blank.

### Matrix Effect

The matrix effect was evaluated both ways, qualitatively and quantitatively.

Qualitative matrix effect was evaluated in six screened interference free plasma lots by postcolumn infusion of analytes using a zero volume tee. One inlet of the tee was connected to the syringe pump for continuous infusion of the analytes. The other inlet was connected to the column outlet, and the remaining tee outlet was connected to the mass spectrometer. After stabilization of response, the processed blank samples were injected to verify ion suppression or ion enhancement as a result of baseline variation. In this study, the mobile phase solution was used as the reference sample to monitor the baseline variation. Similar baseline without any suppression or enhancement at RT of analyte was observed in both samples i.e., extracted blank sample and mobile phase. Therefore, it was concluded that there was no significant matrix effect in the method.

For quantitative evaluation of matrix effect, the peak area response of analyte and IS from aqueous samples (AQS) (representing 100% recovery at LQC and HQC levels) were compared to the extracted blank post-spiked with AQS LQC and AQS HQC, respectively.

Six normal plasma lots, three lipemic plasma lots, and three hemolyzed plasma lots were respectively used to process two replicates of blank samples. The processed blank samples from each of the plasma lot were respectively reconstituted from AQS LQC and AQS HQC to prepare postspiked matrix effect samples. The matrix effect samples were compared with six replicates from each of the AQS LQC and AQS HQC.

The following formula was used to calculate the matrix effect:

$$\begin{array}{l} \begin{array}{l} \text{Matrix factor}=(\text{response in the presence of matrix ions})/\\ \;(\text{response in the absence of matrix ions}) \end{array}\\ \%\text{ Matrix effect}=(1-\text{ mean of matrix factor})\times 100 \end{array}$$

### Precision and Accuracy

To evaluate the precision of the assay, the percent coefficient of variation was calculated at the concentration of LLOQ QC, LQC, MQC, and HQC. The ratio of the calculated mean values at the above four different concentrations to their respective nominal values was used to determine the accuracy of the assay. The goodness of fit analysis was determined using the data of three precision and accuracy batches. The weighing factor, 1/*x* and 1/*x* ^2^, were used to back-calculate the concentrations of CC standards for finding the best fit for regression. The regression equation with a weighting factor of 1/*x* ^2^ gave the best fit for the concentration-detector response relationship for OLM and HCTZ, and linearity was hence calculated.

### Stability

Stock solution and stock dilution stabilities were assessed after 46 h and 45 h at room temperature, respectively. Stock solution and stock dilution stabilities for both analytes and their respective IS were evaluated after 11 days in refrigerator.

Photo-degradation test of OLM, HCTZ, OLM D_6_, and HCTZ ^13^C D_2_ was performed after storage of stock solution in dark and light. Two aqueous mixtures (one from the stability stock solution and another from fresh stock solution [comparison stock]) were prepared for all the aqueous related stability studies. From each of the two aqueous mixtures (stability stock and comparison stock), six replicates were injected. A correction factor was used as follows to correct the response of the stability sample:

$$\begin{array}{l} \text{Correction factor}=(\text{concentration of fresh stock})/\\ \;(\text{concentration of stability stock})\\ \text{Corrected response}=\text{stability stock response}\times \text{correction factor}\\ \;\%\text{ Change}=([\text{mean response of comparison samples }-\;\\ \text{ mean corrected response of stability samples}]/\\ \;[\text{mean response of comparison samples}])\times 100 \end{array}$$

Bench top stability was assessed by using six replicates of LQC and HQC stored at room temperature for 18 h. The extended bench top stability was determined at each step of extraction by assessing the stability of OLM and HCTZ.

After five freeze-thaw cycles, the stability OLM and HCTZ were also determined in the plasma samples. Six replicates of LQC and HQC samples were used for evaluation of freeze-thaw stability. Long-term stability after storage of LQC and HQC spiked plasma samples was assessed at −65°C ± 10°C for 85 days.

The dry extract stability was determined by processing the six sets of LQC and HQC, stored without reconstitution at −20°C ± 5°C. Similarly, the assessment of wet extract stability was carried out by processing the six sets of LQC and HQC, stored at 2–8°C after reconstitution. The wet extract and dry extract stabilities were analyzed after storing samples for 77 h. Fresh stock solutions were used to prepare the six sets of comparison QC’s (freshly spiked LQC and HQC). The % change between the stability QCs and comparison QCs was calculated by analysis of all stability QCs against the freshly spiked CC standards.

### Blood Stability

Blood stability was assessed by spiking aqueous dilutions in blood to explicate the stability of the analytes in blood. Six QC samples (stability samples) were prepared for OLM and HCTZ in blood by spiking aqueous dilution of MQC and HQC, respectively, and keeping in wet ice for 2 h. Freshly spiked QC samples (comparison samples) were also prepared in a similar way. All the stability and fresh QC samples were centrifuged at 4°C for 15 min at 4,000 rpm to separate blood and plasma. Then, plasma samples were processed and analyzed as per the method described above.

The % change between stability samples and comparison samples was calculated as per the following:

$$\begin{array}{c} \text{\% Change = }([\text{mean area ratio of stability samples }-\\ \text{ mean area ratio of comparison samples}]/\\ \text{ [mean area ratio of comparison samples])}\times \text{100} \end{array}$$

### Recovery and PID

Preparation of aqueous recovery samples was carried out by adding 12 μl each from respective QC dilutions (LQC, MQC, and HQC) of OLM and HCTZ; 200 μl of IS dilution (OLM D_6_–2,000.00 ng/ml and HCTZ ^13^C D_2_-2,800.00 ng/ml) and 2,176 μl of mobile phase (representing 100% recovery). The so-prepared aqueous QC samples of OLM and HCTZ were compared with six sets of extracted LQC, MQC, and HQC samples. Similarly, IS recovery was also calculated at levels of LQC, MQC, and HQC.

$$\begin{array}{l} \%\text{ Recovery}=([\text{mean peak area response of extracted sample})/\\ \;(\text{mean peak area response of unextracted sample}])\times 100 \end{array}$$

The effect of PIDs–namely, caffeine, ibuprofen, acetylsalicylic acid, and paracetamol was evaluated by the spiking of the concentration of the drugs at their respective *C*_max_ concentration (maximum/peak plasma concentration achieved by a drug in a specified compartment or test area of the body after the first administration of the drug and before the second dose) in one blank sample and triplicates of LLOQ.

### Robustness and Ruggedness

Robustness was assessed by making slight variations in column temperatures (33°C and 37°C), mobile phase flow rate (0.780 ml/min and 0.820 ml/min), mobile phase compositions (methanol and buffer solution A in the ratio 82:18 and 78:22, v/v), and pH of buffer solution A (approx. 5.65 and at approx 5.35). All CC and QCs fulfilled the acceptance criteria in all abovementioned conditions demonstrating the robustness of the method. To evaluate the ruggedness, a precision and accuracy batch was processed by a different analyst using a different column and different sets of solutions. The results for this batch were again found to be acceptable.

### Dilution Integrity

The dilution integrity experiment was performed for the sample at concentration of 1.7 times of ULOQ, which was named as dilution QC (DIQC). The DIQC samples were further diluted with interference-free plasma for 1/2 and 1/5 dilutions for determining the dilution integrity of samples.

### Bioequivalence Study

The newly developed method was applied to compare the bioequivalence of the test formulation (OLM medoxomil/HCTZ; 40 mg/25 mg film-coated tablet) with the reference formulation (40 mg/25 mg film-coated tablet) in adult, healthy human volunteers under fasting conditions. Plasma samples of subjects completing the entire clinical study were analyzed. Inclusion criteria comprised age (18–45 years) and body mass index i.e., weight in kg/height in meters (18.5–24.9), with normal electrocardiogram and without any abnormalities on physical examination and laboratory tests. The exclusion criteria comprised hypersensitivity known for OLM and HCTZ, psychosis, smoking, alcoholism, diabetes, or any other diseases which could compromise the gastrointestinal, hemopoietic, hepatic, renal, cardiovascular, and central nervous or respiratory systems. In addition, all the procedures of the study were based on the International Conference on Harmonization Guidelines (Guideline for good clinical practice ICH E6 (R1), 1996). The blood samples were collected using K_2_EDTA vacutainers at the following time points: pre-dose and postdose at 0.25, 0.50, 0.75, 1.00, 1.25, 1.50, 1.75, 2.00, 2.25, 2.50, 2.75, 3.00, 3.50, 4.00, 6.00, 8.00, 10.00, 12.00, 18.00, 24.00, 36.00, 48.00, and 72.00 h. The subjects successfully completing periods I and II of the study were considered for pharmacokinetics evaluation. Plasma was separated out immediately after blood sample collection by centrifugation at 4°C for 10 min at 4,500 rpm. Plasma samples were stored in deep freezer at −65°C ± 10ºC until further assays. After completion of the studies, the incurred sample re-analysis was also performed to determine any metabolic change/instability in the plasma samples.

### Incurred Sample Reanalysis

After completion of initial analysis of all subject samples, the incurred sample reanalysis was also performed as per the latest regulatory requirement of bioequivalence studies. A total of 112 incurred samples i.e., two samples from each period of all subjects (one near to *C*_max_ and another one approximately three times of LLOQ concentration from termination phase) were randomly selected and analyzed.

## Results and Discussion

### LC–MS/MS Parameters

**Figure 1:**
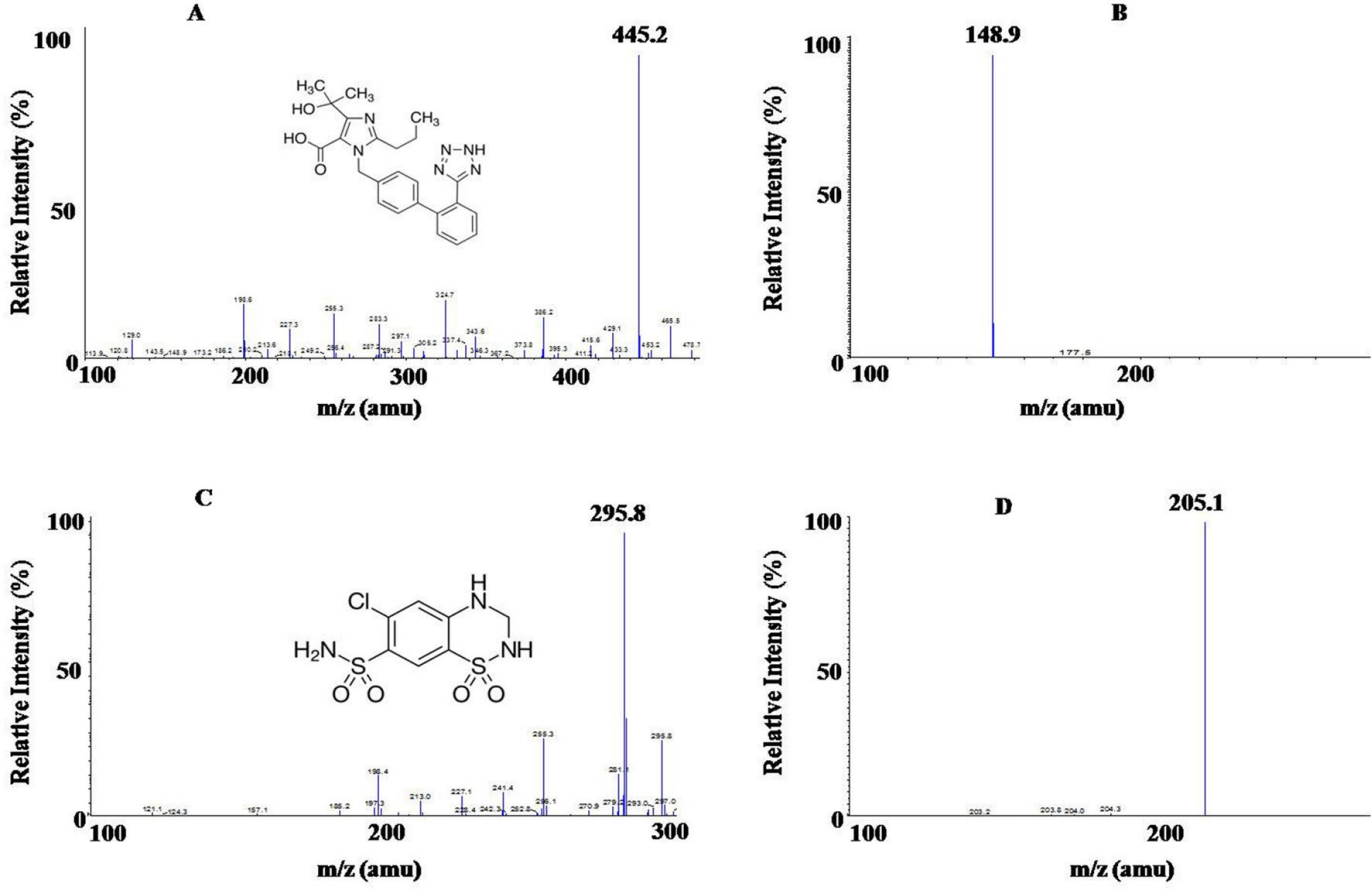
**(A)** Molecular structure of Olmesartan (OLM) OLM with parent ion scan m/z 445.2 amu and **(B)** product ion scan m/z 148.9 amu; **(C)** Molecular structure of hydrochlorothiazide (HCTZ) with parent ion scan m/z 295.8 amu, **(D)** product ion scan m/z 205.1 amu.

Since OLM D_6_ and HCTZ ^13^C D_2_ differ from their respective analytes only in terms of having different isotopic atoms, therefore, they were expected to display nearly similar chromatographic behavior. The respective RT were found to be 1.51 ± 0.3 min for OLM, 1.51 ± 0.3 min for OLM D_6_, 2.11 ± 0.3 min for HCTZ, and 2.11 ± 0.3 min for HCTZ ^13^C D_2_. Furthermore, recovery of OLM D_6_ and HCTZ ^13^C D_2_ is also similar to that of OLM and HCTZ, respectively. High ionization efficiencies were obtained in negative ion mode with ESI for both analyte and respective IS. The outstanding sensitivity of the method was primarily due to these high ionization efficiencies. The structure and mass spectra of the parent and product ions of OLM (mol. Wt. 446.50) and HCTZ (mol. Wt. 297.74) are shown in **Figure 1**. The optimized Electrospray Ionization Tandem Mass Spectrometry (ESI-MS/MS) compound parameters and source dependent parameters for OLM, HCTZ, and IS (OLM D_6_ and HCTZ ^13^C D_2_) are given in **Tables 1** and **2**.

**Table 1:** ESI-MS/MS compound parameters for OLM, OLM D_6_, HCTZ, and HCTZ ^13^C D_2_.

Compound parameters	OLM	OLM D_6_	HCTZ	HCTZ ^13^C D_2_
Q1 mass (precursor ion)	445.20 amu	451.40 amu	295.80 amu	298.90 amu
Q3 mass (product ion)	148.90 amu	154.30 amu	205.10 amu	206.30 amu
Declustering potential (V)	−70	−70	−85	−85
Entrance potential (V)	−10	−10	−10	−10
Collision energy (V)	−33	−48	−30	−30
Collision cell exit potential (V)	−10	−10	−10	−10
Dwell time (milliseconds)	200	200	200	200

**Table 2:** ESI-MS/MS source parameters for OLM, OLM D_6_, HCTZ, and HCTZ ^13^C D_2_.

Source parameters	Values
Nebulizing gas	40 psi
Auxiliary gas	40 psi
Curtain gas	35 psi
Collision-activated dissociation (CAD)	6
Ion source (voltage)	−2,000
Ion source temperature	550°C
Interface heater (ihe)	ON

### Selectivity and Matrix Effect

**Figure 2:**
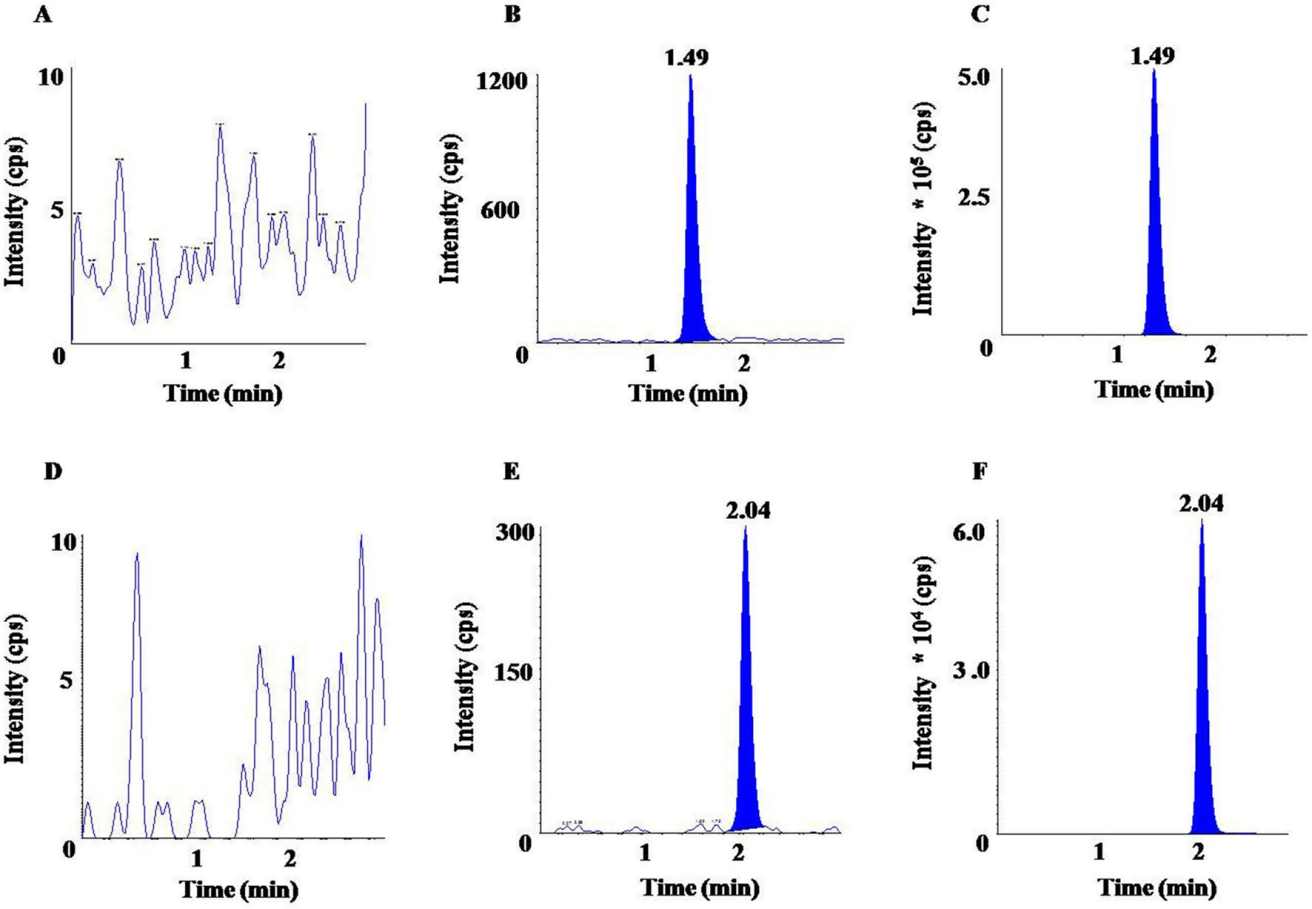
Chromatograms of OLM for **(A)** blank, **(B)** LLOQ (lower limit of quantification), **(C)** upper limit of quantification (ULOQ); and chromatograms of HCTZ for **(D)** blank, **(E)** LLOQ, **(F)** ULOQ.

The chromatograms represented in **Figure 2** clearly showed absence of sharp peaks in blank samples (**Figures 2A, D**) at the respective RTs of OLM (1.49 min) and HCTZ (2.04 min), whereas a sharp and symmetric peak was observed for LLOQ and ULOQ concentrations of OLM (**Figures 2B, C**) and HCTZ (**Figure 2E, F**). Thus, it can be inferred that there was no significant interference due to endogenous substances at the respective RT of the analyte and IS in normal, hemolyzed, as well as in lipemic plasma. The matrix factor variability, represented here as % CV of matrix factor was obtained as 3.99% (HQC) and 4.00% (LQC) for OLM, 3.42% (HQC) and 4.32% (LQC) for OLM D_6_, 4.65% (HQC) and 9.51% (LQC) for HCTZ, and 3.83% (HQC) and 5.48% (LQC) for HCTZ ^13^C D_2_. The variability of IS-normalized matrix factor similarly represented here as % CV of matrix factor was found to be 2.08% (HQC) and 5.55% (LQC) for OLM and 5.08% (HQC) and 8.31% (LQC) for HCTZ. The % matrix effect ranged from -1.95% (HQC) to 5.91% (LQC) for OLM and from −5.03% (HQC) to 3.43% (LQC) for HCTZ. The results obtained in this study were found to fulfill the acceptance criteria, which indicated that plasma matrix did not result in any ion enhancement or suppression.

### Linearity and Sensitivity

Substituting the respective values in the typical equations of CC, the correlation coefficient (*r* ^2^) was obtained as follows:

For OLM,

$$\text{y}=0.0012627\times +0.002445;\;{\text{r}}^{2}=0.9987$$

For HCTZ,

$$\text{y}=0.005392\times +–0.002463;\;{\text{r}}^{2}=0.9981$$

Here, “*y*” represents the analyte/IS peak area ratio and “*x*” represents the plasma concentration of the analyte.

Therefore, for both the analytes, the correlation coefficient (*r* ^2^) was found to be greater than 0.98 over the linearity range, viz. 5.002 ng/ml to 2,599.934 ng/ml for OLM and 3.005 ng/ml to 499.994 ng/ml for HCTZ.

The precision and accuracy for OLM at LLOQ were found to be 3.23% and 98.54%, respectively. For HCTZ, the precision and accuracy at LLOQ were obtained as 1.49% and 97.14%, respectively. This high degree of precision and accuracy revealed a remarkable sensitivity of the method.

As per the USFDA, the lower limit of quantification should be lower than 5% of reported *C*_max_. The *C*_max_ value was reported to be ≈1,350 ng/ml for OLM (for 40 mg single dose as per Kumar et al., 2014) and ≈216 ± 54 ng/ml for HCTZ (for 25 mg dose according to Gao et al., 2010). In our study, we achieved the lower limit of quantification at 5.002 ng/ml for OLM and 3.005 ng/ml for HCTZ (which is still lower than 5% of Cmax), with signal to noise ratio of >5 and precision and accuracy of <20% and ±20%, respectively.

The back-calculated concentrations of CC standards for OLM and HCTZ are summarized in **Table 3**, and **Table 4** represents the data for intraday and interday precision and accuracy. As our results remained within the acceptable limits, it certainly demonstrated that our method was fairly precise and accurate to be further used in pharmacokinetic analysis.

**Table 3:** CC Standards Data (STD) for three independent Precision and Accuracy (PA) Batches.

CC STD Data for three independent PA Batches for OLM											
CC Standards	STD A	STD B	STD C	STD D	STD E	STD F	STD G	STD H	Slope	Intercept	*r* ^2^
**Nominal concentration (ng/ml)**	5.00	10.00	299.51	599.02	1,198.05	1,633.35	2,129.56	2,599.93			
**Observed concentration PA Batch 1**	5.10	9.55	317.47	612.47	1,209.66	1,591.30	2,081.19	2,546.98	0.001627	0.002445	0.998
**Observed concentration PA Batch 2**	4.87	10.49	314.80	621.56	1,188.31	1,597.46	2,050.82	2,482.02	0.001642	0.001826	0.998
**Observed concentration PA Batch 3**	4.80	10.77	314.15	629.67	1,244.38	1,614.62	1,987.46	2,344.75	0.001593	0.001971	0.995
**Observed mean**	4.92	10.27	315.47	621.23	1,214.12	1,601.13	2,039.82	2,457.92	0.001621	0.002081	0.997
**% CV**	3.23	6.21	0.56	1.38	2.33	0.75	2.34	4.20			
**% Bias**	−1.46	2.74	5.33	3.71	1.34	−1.97	−4.21	−5.46			
**N**	3	3	3	3	3	3	3	3	3	3	3
CC STD data for three independent PA batches for HCTZ											
CC Standards	STD A	STD B	STD C	STD D	STD E	STD F	STD G	STD H	Slope	Intercept	*r* ^2^
**Nominal concentration (ng/ml)**	3.00	6.01	62.59	125.19	250.39	326.80	425.76	499.99			
**Observed concentration PA Batch 1**	3.03	5.91	57.23	126.91	254.08	323.31	435.26	525.01	0.005392	−0.002463	0.998
**Observed concentration PA batch 2**	3.11	5.58	58.83	125.37	245.94	350.17	418.62	527.45	0.005590	−0.001123	0.996
**Observed concentration PA batch 3**	3.11	5.56	62.58	119.27	257.55	321.92	427.82	532.74	0.005421	−0.001569	0.997
**Observed mean**	3.09	5.69	59.55	123.85	252.52	331.80	427.24	528.40	0.005468	-0.001718	0.998
**% CV**	1.49	3.48	4.61	3.26	2.36	4.80	1.95	0.75			
**% Bias**	2.86	−5.32	−4.87	−1.07	0.85	1.53	0.35	5.68			
**N**	3	3	3	3	3	3	3	3	3	3	3

**Table 4:** Intraday and interday precision and accuracy for the detection of OLM and HCTZ in human plasma.

Nominal concentration (ng/ml)	OLM				HCTZ			
	LLOQ QC	LQC	MQC	HQC	LLOQ QC	LQC	MQC	HQC
	5.006	13.906	1,198.829	2,131.831	3.008	8.076	252.367	419.392
**No of replicates for intraday (** ***n*** **)**	6	6	6	6	6	6	6	6
**Observed mean**	4.953	13.055	1,195.508	2,079.473	3.209	8.576	259.416	435.590
**% CV**	4.58	10.38	3.67	3.24	5.67	11.81	4.99	3.57
**% Bias**	−1.06	−6.12	−0.28	−2.46	6.68	6.19	2.79	3.86
**No of replicates for interdays (** ***n*** **)**	18	18	18	18	18	18	18	18
**Observed mean**	4.755	13.507	1,198.816	2,108.060	3.068	8.324	258.846	435.349
**% CV**	9.02	6.65	3.15	3.07	8.21	8.12	4.52	3.32
**% Bias**	-5.01	−2.87	0.00	−1.12	1.99	3.07	2.57	3.80
**% Total error**	14.04	9.52	3.15	4.19	10.21	11.19	7.08	7.13

### Recovery

Due to the substantial differences in the physiochemical properties i.e., partition coefficient (log *p*) and dissociation coefficient (pKa) between OLM (log *p* = 0.73, pKa = 4.3) and HCTZ (log *p* = −0.07, pka = 7.9), it was tricky to extract this combination of drugs with the LLE. In our study, we succeeded in extraction of these two drugs by addition of buffer solution into the plasma sample and achieved a comparable recovery of OLM and HCTZ with their respective IS, well suited for pharmacokinetic studies.

The mean percentage recovery of OLM was 66.96% with a precision of 7.35% and that for OLM D_6_ was 68.01% with a precision of 7.77%. The mean percentage recovery of HCTZ was observed to be 81.33% with a precision of 3.85% whereas that for HCTZ ^13^C D_2_ was 82.83% with a precision of 4.34%. The observed percentage of recovery and precision indicated that our LLE procedure is highly consistent and efficient for extraction of both analyte and IS from human plasma. We could thus extract both analytes and their IS consistently, without the matrix interference by LLE.

### Stability and Other Parameters

The stability experiments performed in this study revealed that no degradation or instability was observed when the samples were stored in the storage conditions as described in **Table 5**. The % CV and % stability obtained from our stability experiments were less than 15%, which was well within the acceptance criteria of 15% for the same. In addition, the results obtained for other parameters of method validation—namely, ruggedness, robustness, dilution integrity, re-injection reproducibility, effect of PIDs, and extended batch verification—were obtained well within the pre-set acceptable criteria of USFDA guidelines. Therefore, this assay demonstrated the suitability for applications in pharmacokinetic studies.

**Table 5:** Stability results of OLM and HCTZ (*n* = 6).

Stability	Analyte	QC level	Mean concentration of comparison samples (ng/ml)	% CV	Mean concentration of stability samples (ng/ml)	% CV	% Stability (% change in Mean)
**Bench top stability** **(18 h)**	OLM	LQC	13.643	4.96	13.874	1.83	1.50%
		HQC	2,098.233	1.65	2,110.802	1.27	0.77%
	HCTZ	LQC	8.122	4.44	8.714	4.89	1.09%
		HQC	422.499	1.11	417.868	2.15	−0.88%
**Freeze-thaw stability** **(5 cycles)**	OLM	LQC	13.643	4.96	13.710	3.39	0.30%
		HQC	2,098.233	1.65	2,102.909	1.32	0.39%
	HCTZ	LQC	8.122	4.44	8.309	4.59	2.76%
		HQC	422.499	1.11	422.405	1.79	0.20%
**Autosampler stability** **(5°C, 80 h)**	OLM	LQC	14.146	6.85	13.935	3.32	−1.80%
		HQC	2,109.196	1.54	2,142.448	3.31	1.62%
	HCTZ	LQC	7.904	3.05	8.346	2.94	5.76%
		HQC	424.849	1.05	430.567	4.41	1.28%
**Dry extract stability (-22°C, 77 h)**	OLM	LQC	14.146	6.85	13.952	6.39	−1.68%
		HQC	2,109.196	1.54	2,117.386	1.64	0.43%
	HCTZ	LQC	7.904	3.05	8.025	4.59	1.69%
		HQC	424.849	1.05	427.844	2.09	0.64%
**Wet extract stability (2–8°C, 77 h)**	OLM	LQC	14.146	6.85	13.965	7.17	−1.58%
		HQC	2,109.196	1.54	2,100.409	1.07	−0.37%
	HCTZ	LQC	7.904	3.05	8.264	4.20	4.72%
		HQC	424.849	1.05	423.634	2.01	0.35%
**Hemolyzed stability**	OLM	LQC	14.146	6.85	13.805	2.28	−2.71%
		HQC	2,109.196	1.54	2,142.737	1.10	1.64%
	HCTZ	LQC	7.904	3.05	8.122	4.51	2.92%
		HQC	424.849	1.05	430.019	3.22	1.15%
**Lipemic stability**	OLM	LQC	14.146	6.85	13.925	5.56	−1.87%
		HQC	2,109.196	1.54	2,075.608	2.25	−1.55%
	HCTZ	LQC	7.904	3.05	8.281	2.96	0.05%
		HQC	424.849	1.05	425.323	1.72	4.94%
**Long-term stability (−65°C ± 10°C, 85 days)**	OLM	LQC	13.546	2.85	13.404	2.48	−1.61%
		HQC	2,107.205	1.88	2,090.491	0.88	−1.08%
	HCTZ	LQC	8.102	3.23	8.536	4.09	5.70%
		HQC	421.334	1.29	414.545	2.29	−1.16%

### Blood Stability

When blood samples were stored in wet ice for 2 h, no degradation was observed which is evident from the results represented in **Table 6**. The % change of the mean area ratio between stability and comparison samples was also found to be well within the acceptance limit.

**Table 6:** Blood Stability results.

Parameters	OLM				HCTZ			
	MQC		HQC		MQC		HQC	
	Stability samples	Comparison samples	Stability samples	Comparison samples	Stability samples	Comparison samples	Stability samples	Comparison samples
**Mean area ratio**	2.23260	2.22022	3.91937	3.86793	1.57117	1.53827	2.63160	2.62385
**% CV**	1.01	1.21	1.79	1.69	3.01	1.71	2.18	2.92
**% Change of mean area ratio**	0.56		1.33		2.14		0.30	

### Dilution Integrity

The precision (% CV) and accuracy (mean % nominal) for OLM were found to be 1.43% and 97.78% for the samples with dilution factor of 1/2 whereas the precision and accuracy were observed to be 2.51% and 101.17% for the samples with dilution factor of 1/5.

Again, the precision (% CV) and accuracy (mean % nominal) for HCTZ were found to be 2.60% and 104.88% for the samples with dilution factor of 1/2 and the precision and accuracy were 2.18% and 109.75% for the samples with dilution factor of 1/5.

### Pharmacokinetic Evaluation

The as developed, validated bioanalytical method was applied in the bioequivalence study conducted on 28 healthy, human subjects for the simultaneous determination of plasma concentrations of OLM and HCTZ. The primary pharmacokinetic parameters viz., maximum plasma concentration (*C*_max_) and area under the plasma concentrations *versus* time curve (AUC) ranging from time zero to last measurable concentration (AUC_0–t_) and extrapolated to infinity (AUC_0-∞_) were compared by an analysis of variance (ANOVA). These pharmacokinetic parameters including 90% confidence interval, power (%), and bioequivalence output for OLM and HCTZ are illustrated in **Tables 7** and **8**, respectively. The secondary pharmacokinetic parameters viz. *T*_max_ (sampling time in h to reach maximum drug concentration), AUC_Extrap_ (percentage of area under plasma concentration extrapolated from AUC_0–t_ to AUC_0-∞_), K_el_ (Elimination rate constant), *T*_1/2_ (drug half life in h), TLIN (time point in h where log-linear elimination phase begins), and LQCT (time in h at which the last concentration above the limit of quantitation occurred) were evaluated for test and reference formulation of OLM and HCTZ, respectively, in**** **Tables 9** and **10**.

**Table 7:** ANOVA analysis of primary pharmacokinetic parameters for OLM after oral administration of single dose of test and reference tablets in human subjects (*n* = 28).

Drug	Statistics	*C*_max_ (ng/ml)	AUC_0–t_ (ng*h/ml)	AUC_0-∞_ (ng*h/ml)
**Test**	Least square mean	7.3461	9.1244	9.2255
	Geometric mean	1,314.4287	8,726.5828	8,845.9674
	% Relative standard deviation (RSD)	377.05807	2,383.67078	2,389.97799
**Reference**	Least square mean	7.3128	9.1202	9.2182
	Geometric mean	1,231.8794	8,425.7292	8,555.2911
	% RSD	345.89734	2,511.06425	2,530.58673
**T/R ratio (%)**		106.70	103.57	103.40
**90% Confidence interval for the ratio of the mean test/reference**		99.60–113.67	98.88–108.85	98.72–108.70
**Power (%)**		99.98	99.99	99.99
**Bioequivalence**		Yes	Yes	Yes

**Table 8:** ANOVA analysis of primary pharmacokinetic parameters for HCTZ after oral administration of single dose of test and reference tablets in human subjects (*n* = 28).

Drug	Statistics	C_max_ (ng/ml)	AUC_0–t_ (ng*h/ml)	AUC_0-∞_ (ng*h/ml)
**Test**	Least square mean	5.1916	6.9223	6.9966
	Geometric mean	167.6787	1,143.0527	1,207.7012
	% RSD	34.05764	226.72581	225.70831
**Reference**	Least square mean	5.1460	6.9695	7.0332
	Geometric mean	165.9303	1,155.9354	1,218.1618
	% RSD	35.15450	210.20325	202.55876
**T/R ratio**		101.05	98.89	99.14
**90% Confidence interval for the ratio of the mean test/reference**		94.69–108.13	95.95–101.97	96.17–102.08
**Power (%)**		99.81	99.75	99.88
**Bioequivalence**		Yes	Yes	yes

**Table 9:** Secondary pharmacokinetic parameters for OLM after oral administration of single dose of test and reference tablets in human subjects (*n* = 28).

Drug	Statistics	*T*_max_ (h)	AUC_Extrap_ (%)	K_el_ (1/h)	*T*_1/2el_	TLIN (h)	LQCT (h)
**Test**	**Mean**	1.954	1.106	0.092	7.951	17.231	45.778
	**% CV**	37.06	46.40	21.34	24.57	32.52	16.32
**Reference**	**Mean**	2.185	1.069	0.089	8.016	18.370	46.222
	**% CV**	31.51	57.24	17.21	20.69	30.05	15.62
**T/R ratio (%)**		89.43	103.46	103.37	99.19	93.80	99.04

T_max_, Sampling time in h to reach maximum drug concentration (C_max_); AUC_Extrap_, Percentage of area under plasma concentration extrapolated from AUC_0–t_ to AUC_0-∞_; K_el_, Elimination rate constant; T_1/2_, Drug half life in h; TLIN, Time point in h where log-linear elimination phase begins; LQCT, Time in h at which the last concentration above the limit of quantitation occurred.

**Table 10:** Secondary pharmacokinetic parameters for HCTZ after oral administration of single dose of test and reference tablets in human subjects (*n* = 28).

Drug	Statistics	*T*_max_ (hr)	AUC_Extrap_ (%)	*K*_el_ (1/hr)	*T*_1/2el_	TLIN (h)	LQCT (h)
**Test**	**Mean**	1.935	7.069	0.078	9.176	11.806	31.111
	**% CV**	46.93	47.60	19.44	15.16	33.08	22.08
**Reference**	**Mean**	1.852	6.121	0.076	9.156	11.704	32.444
	**% CV**	47.55	43.57	13.75	11.95	26.85	22.51
**T/R ratio (%)**		104.48	115.49	102.63	100.22	100.87	95.89

**Figure 3:**
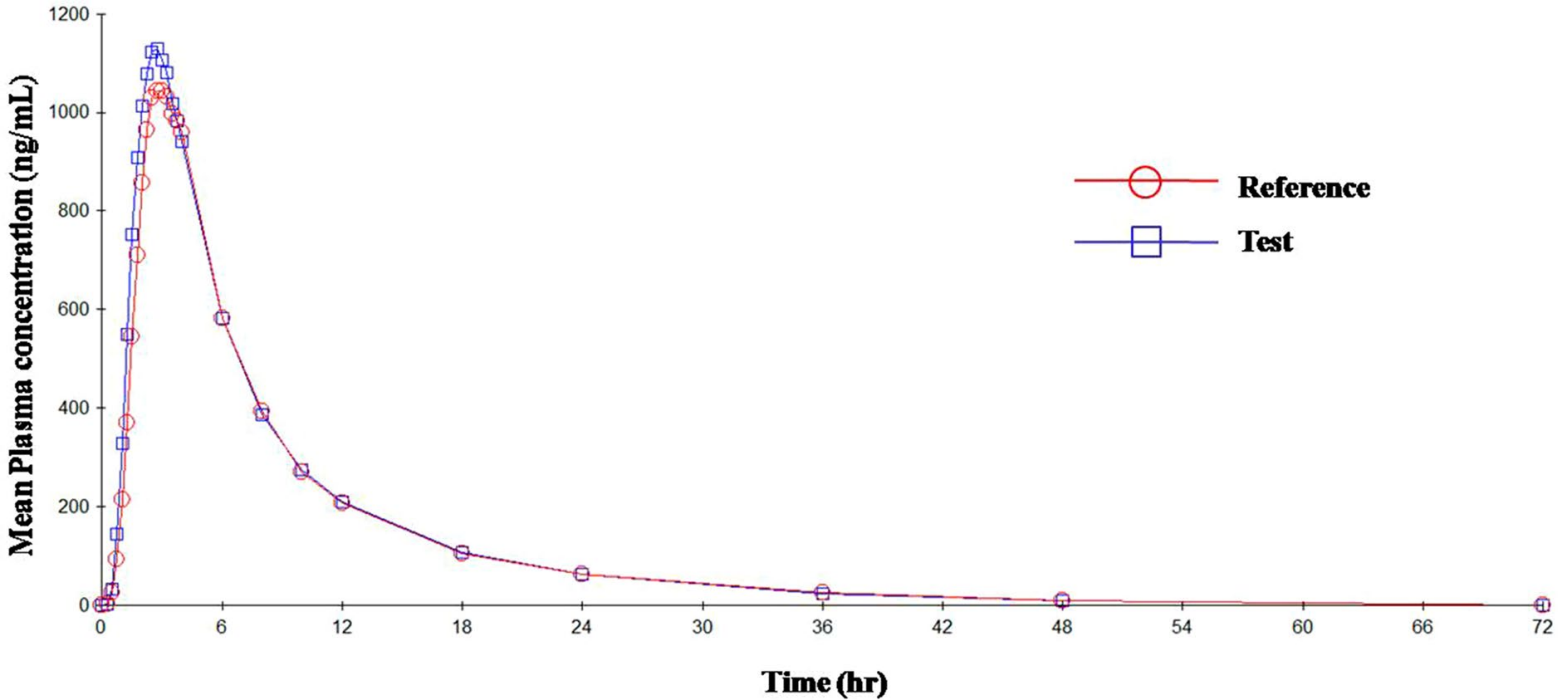
Mean plasma concentration vs. time curve for OLM after administration of single dose of test and reference tablets.

**Figure 4:**
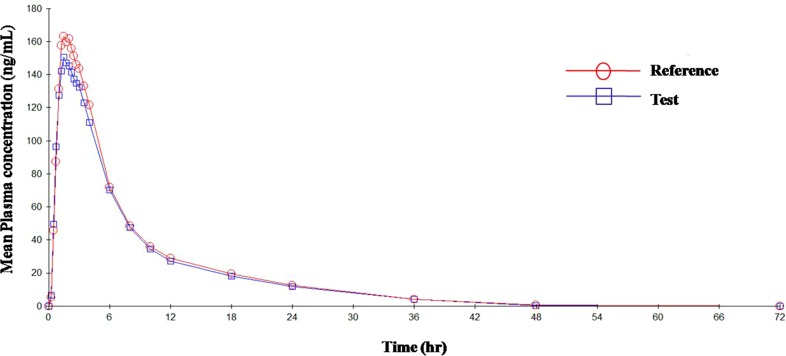
Mean plasma concentration vs. time curve for HCTZ after administration of single dose of test and reference tablets.

The mean plasma concentrations versus time curve for the two drugs are given in **Figures 3** and **4**. In accordance to regulatory requirements, bioequivalence is concluded in a study if the 90% confidence interval of the adjusted geometric mean ratios for *C*_max_ and AUC lie within the predetermined range of 80–125%. In our study, the 90% confidence interval of the log transformed geometric mean for test to reference ratio was found to be well within the acceptance criteria of 80–125% for AUC_0‐∞_ and *C*_max_. Hence, it was concluded that the test product was bioequivalent to the reference product.

### Incurred Sample Reanalysis

All incurred sample concentration was found to be within the ±20% of their initial concentration, which proved that newly developed, validated method is competent for precise, accurate, and reproducible quantification of OLM and HCTZ in human plasma and any metabolic change/instability in the plasma samples was not found under these validated conditions.

## Conclusion

Limitations exist in the conventional methods based on spectrophotometer, HPLC, and HPTLC, which were used for the quantification of drugs. Spectrophotometer can only be used if the substance under analysis has significant absorbance in the UV-visible range, but unfortunately, all compounds do not have this property. Sometimes, other molecules present in the sample can also absorb at the wavelength under consideration, which can interfere leading to an incorrect estimation of the concentration. Most of the reported HPLC and HPTLC methods have long run times; therefore, they are considered inappropriate for routine use in high-throughput clinical studies. HPLC and HPTLC methods were developed for the pharmaceutical dosage forms, so they are not suitable for the biological samples. LC-MS/MS offers the advantage of higher sensitivity and specificity and significantly shorter analysis time over these relatively old analysis techniques and has therefore emerged as the first choice of researchers for the estimation of drugs in biological samples.

The conducted study could successfully develop a highly sensitive and selective method for the simultaneous quantitative determination of OLM and HCTZ in human plasma using LC–MS/MS with turbo-ion spray in negative ion mode through LLE method for sample preparation. The method was validated in accordance with the USFDA guidelines. The novel LLE method was developed for the sample pre-treatment. Sample pre-treatment is the major part in the analysis of drugs from biological samples, because more than 50% of cost, labor participation, and errors are associated with the sample pre-treatment. Therefore, it is always advisable to make the sample pre-treatment process as simpler, cost-effective, and robust as possible, without compromising on the selectivity, sensitivity, precision, and accuracy. This method was found to be appropriate for further pharmacokinetic, bioavailability and bioequivalence studies. As compared to previously published methods, this method has the advantage of shorter chromatographic run time (3.0 min) resulting in high-throughput sample analysis. The hemolysis, lipemic stability, blood stability, dilution integrity analysis, and incurred sample re-analysis of OLM and HCTZ were also evaluated for the first time, and it was found that drug was stable in blood, which is a crucial factor for bioequivalence and pharmacokinetic studies. All these parameters were found well within acceptance limits which demonstrated the suitability of this newly developed method for the high-throughput sample analysis in routine, clinical, and pharmacokinetic studies. The applicability of this method for further pharmacokinetic studies was successfully demonstrated through a bioequivalence study conducted on healthy human subjects.

In order to improve the chromatography, the method was applied on the advanced version of the instrument (LC-MS/MS, API 4000). For better analytical results, structural analogues were replaced by respective deuteriated standards. To summarize, this as developed novel and high-throughput LLE bioanalytical method has a novel and substantial innovative value with the benefits of lower cost, robustness, good extraction efficiency, and environmental friendly with lesser consumption of organic solvents, shorter analysis time, and simpler procedure. Since the analysis cost directly impacts the drug development process and overall incurred cost of the final product, therefore, this newly developed method is likely to help in reducing the drug development budget.

## Ethics Statement

This study was carried out in accordance with the recommendations of the Basic Principles defined in US 21 CFR part 320, the ICH guidelines for Good Clinical Practice and principles enunciated in Declaration of Helsinki. The study protocol and informed consent forms (ICF) were approved in 2013 by Hippocrates Independent Ethics Committee. All subjects gave written informed consent in accordance with the Declaration of Helsinki after being informed of the purpose and risks of the study.

## Author Contributions

The experiments were performed by AK. The experiments were designed by all authors. Manuscript preparation and finalization was carried out by AK and TP.

## Conflict of Interest Statement

The authors declare that the research was conducted in the absence of any commercial or financial relationships that could be construed as a potential conflict of interest.
